# Shape-anisotropic enhanced damping in CoZr periodic arrays of nanohill structure

**DOI:** 10.1186/1556-276X-8-284

**Published:** 2013-06-12

**Authors:** Fenglong Wang, Gaoxue Wang, Changjun Jiang, Desheng Xue

**Affiliations:** 1 Key Lab for Magnetism and Magnetic Materials of the Ministry of Education, Lanzhou University, Lanzhou 730000, People’s Republic of China

**Keywords:** Anodized aluminum oxide, Thin films, Nanohill structure

## Abstract

The preparation of CoZr nanostructure films to replicate the order of anodized aluminum oxide template with barrier layer was described. Coercivity and in-plane magnetic anisotropy were increased with the increase of oblique sputtering angle. Resonance frequency and damping factor had the same tendency also. Note that larger damping factor in nanostructure films was observed compared with that of continuous films in Si substrate, which was induced by magnetic anisotropy distribution particularly with a significant out-of-plane contribution due to the competition of shape anisotropy.

**PACS:**

75.75.-c, 75.70.-i, 07.57.Pt

## Background

It was known that working frequency is moving to the gigahertz band region for applications such as magnetic recording heads, wireless inductor cores, and microwave noise filters [1]. It requires the development of a soft magnetic film with high resonance frequency and high permeability [2,3]. In order to solve the expanded electromagnetic interference problems, many researchers begin to focus on the enhancement of microwave absorption [4]. Magnetic thin film application is based on the analysis of the dynamic magnetic or magnetization process, which is subjected to an effective magnetic anisotropy field *H*_eff_ as given by the Landau-Lifshitz-Gilbert (LLG) equation [5] and resonance frequency *f*_r_[6]

(1) $$\frac{\mathrm{dM}}{\mathrm{dt}}=-\gamma \left(M\times H_{\mathrm{eff}}\right)+\frac{\alpha }{M_{s}}M\times \frac{\mathrm{dM}}{\mathrm{dt}}$$

(2) $$f_{r}=\frac{\gamma }{2\pi }\sqrt{M_{s}H_{\mathrm{eff}}}$$

where *M*_s_ represents saturation magnetization, *H*_eff_ is the anisotropy effective field, *γ* is the gyromagnetic factor, and *α* is the damping constant. From Equations 1 and 2, it can be seen that magnetic anisotropy and saturation magnetization are the two key material parameters which determine the magnetic properties of the magnetic film. The resonance frequency can be regulated through magnetic anisotropy. Generally, magnetic anisotropy is affected by many factors, such as demagnetization energy from the sample’s shape or microstructure [7], magneto-crystalline energy from the material’s crystal symmetry [8], magneto-elastic interactions from the stress state of the sample [9], single-ion anisotropy or pair order from chemical short-range order effect [10], exchange anisotropy from the ferromagnetic-antiferromagnetic coupling [11], etc. For thin films, in-plane uniaxial anisotropy determines microwave magnetic properties. Usually, uniaxial magnetic anisotropy is induced by many methods, for example, controlling the sputtering angle [12,13], changing the target-substrate distance [14], controlling the stress [9,15], using nanowire arrays [16], etc.

Ordered magnetic nanostructures, composed of arrays of different kinds of magnetic elements arranged in a periodic fashion, have attracted increasing attention in recent years [17,18]. Shape anisotropy was introduced with spatial dependence on a very small length scale when a periodic nanostructure is defined in a continuous magnetic thin film. The rapid advance in the fabrication of nanostructures, with controlled submicron size and shape offered by modern lithography techniques like ion or electron beam lithography, has triggered increased research on magnetic nanostructures (dots, stripe, or antidots) with a variety of shapes [19-21]. Anodized aluminum oxide (AAO) template with a high areal density [22,23] (up to 1,011 pores/cm^2^) and narrow size distribution over a large area has received much attention because of its simple and inexpensive control of structural parameters and excellent thermal and mechanical stability.

Various routes have been proposed to replicate the ordering of AAO where the final replicated nanostructures consist of highly ordered glassy antidots, nanowire, etc. In these nanostructured materials, large coercivity is induced due to strong shape anisotropy, which have attracted a great deal of interest owing to their potential applications as optoelectronics, data storage materials, surface modifiers with specific wetting behavior, etc. [24]. However, in order to apply magneto-electronic devices in the gigahertz region, a soft magnetic film with low coercivity and in-plane uniaxial anisotropy is developed. Therefore, in the present work, we use an AAO nanostructure with barrier layer as a substrate. CoZr nanohill structured magnetic film (approximately 25 nm) has been sputtered onto a barrier layer of AAO by oblique sputtering. Oblique sputtering would induce in-plane uniaxial anisotropy [25] and increase shape anisotropy. We investigated static and dynamic magnetic properties of CoZr nanostructured films with various oblique sputtering angles and obtained adjustable resonance frequency and linewidth.

## Methods

The annealed aluminum foil (99.95%) was used to prepare the single anodic alumina template (AAO). Two-step oxidation was used to obtain the anodic alumina template. At the first step, Al was anodized in 0.3 M oxalic acid at 40 V for 1 h. Then the alumina from the first step was etched away by an alumina etchant (chromic acid and phosphoric acid) at 60°C for 30 min. At the second step, the oxidation was similar to the first step, but the oxidation time was 8 h.

**Figure 1:**
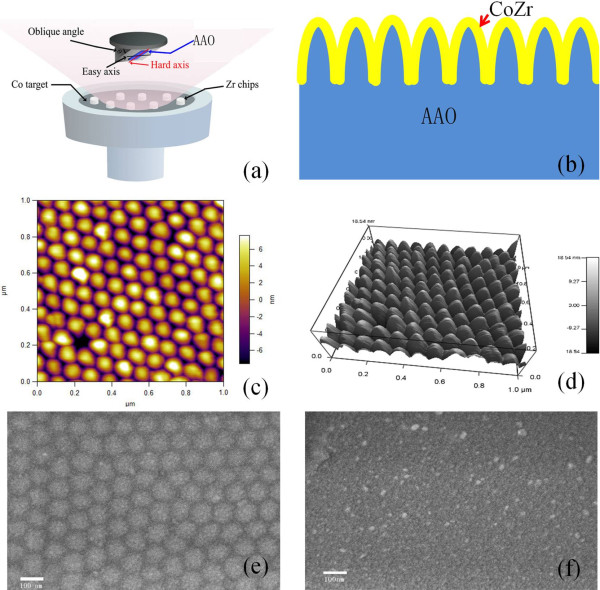
**The nanostructured thin film. (a)** Schematic illustration of the sputtering arrangement. **(b)** Schematic of the layer structure. **(c** and **d)** AFM image of the barrier layer surface of the AAO template. SEM images of the **(e)** 0° and **(f)** 60°samples.

CoZr soft magnetic thin film was prepared by radio frequency sputtering onto the single anodic alumina template with a background pressure lower than 6.0 × 10^−5^ Pa, and a 0.2-MPa pressure of argon was used in the sputtering. A Co target, 70 mm in diameter and 3 mm in thickness, on which eight Zr chips were placed in a regular manner, was used as Figure 1a shows. The sputtering angle of the film was from 0° to 60°, every 20°. Growth rate at different oblique angles was different; we kept all samples 50-nm thick with adjusting of the sputtering time. Figure 1b shows the schematic of the layered structure. The surface morphology of the arrays was investigated with an atomic force microscope (AFM; MFP-3D(TM), Asylum Research, Goleta, CA, USA) and scanning electron microscope (SEM; Hitachi S-4800, Tokyo, Japan). The static magnetic properties of the samples were measured using a vibrating sample magnetometer (VSM). Out-plane ferromagnetic resonance (FMR) measurements were performed with a JEOL JES-FA 300 spectrometer (JEOL, Tokyo, Japan; X-band at 8.969 GHz). The microwave permeability measurements of the films were performed using a vector network analyzer (PNA E8363B) with a microstrip method.

## Results and discussion

Figure 1c,d shows the AFM surface morphology of the barrier layer in the anodic alumina oxide template. From the figure, the barrier layer surface presented smooth mountains with heights of around 10 nm. In the template production process, the process parameters of template projection were oxidation voltage and electrolyte concentration. With the increase of oxidation voltage, the diameter of the projection increases; when electrolyte concentration increases, the current density increases, and there is increase in the diameter of the projection. The reason for the projections formed could be explained by the electric field under the support of the template oxidation process dissolution model [26]. The charge was the most concentrated at the bottom of the holes, and dissolution rate was the fastest. Figure 1e,f shows the SEM micrographs of the 0° and 60° samples. As shown from the figure, the sample of the oblique 0° kept the nanohill shape from replicating the order of an anodized aluminum oxide template with barrier layer; however, this nanostructure disappeared with oblique sputtering, as shown Figure 1f.

**Figure 2:**
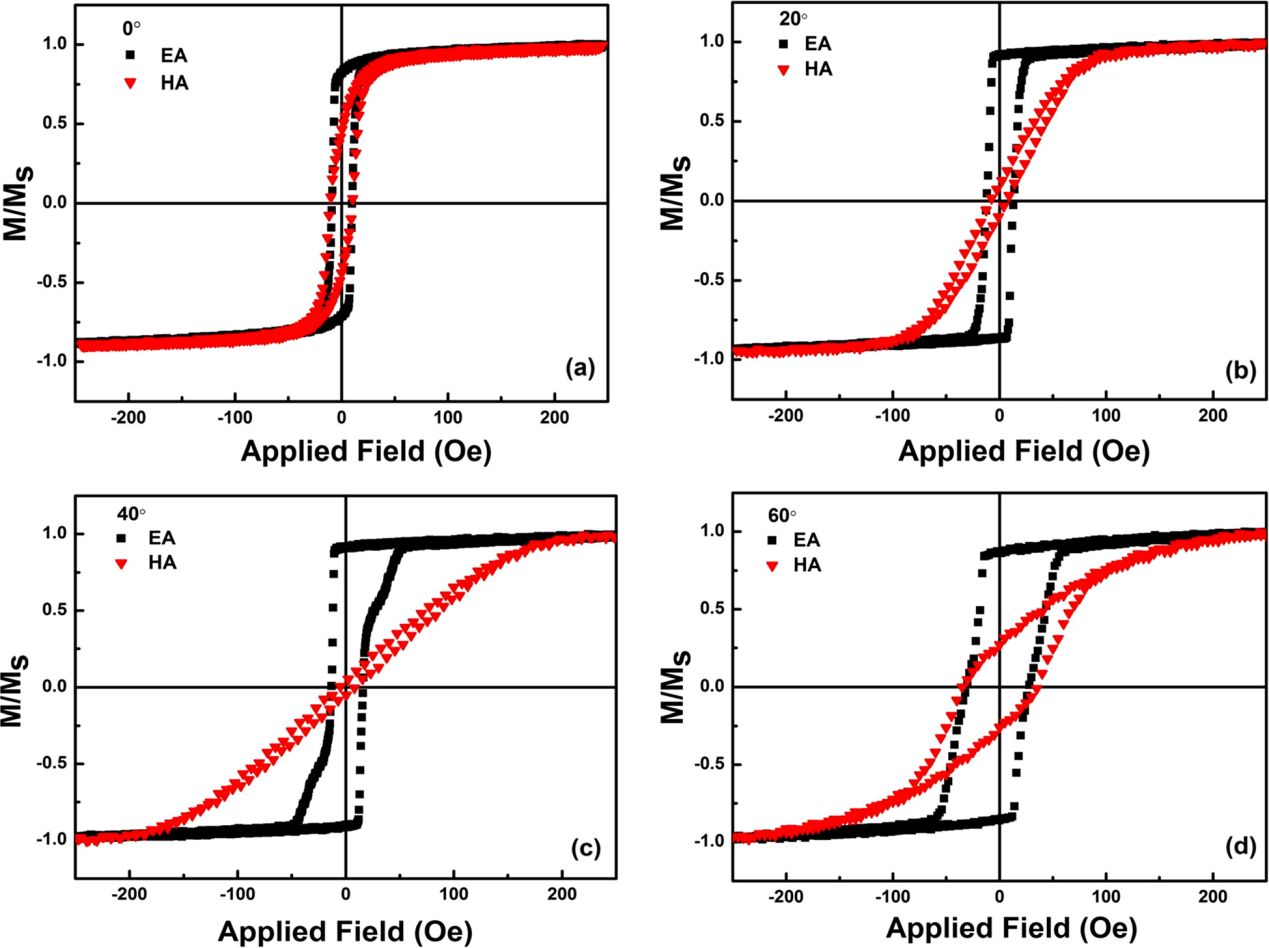
***M*/*M***_**s **_**loops along both easy axes and hard axes. (a)** 0°, **(b)** 20°, **(c)** 40°, and **(d)** 60° samples.

Figure 2 shows the magnetic hysteresis loops of the as-deposited CoZr thin films with oblique sputtering angle from 0° to 60° measured at room temperature, where the magnetic field was applied on the film plane. Note that for the sample with oblique sputtering angle of 0°, the results of the static magnetic measurements revealed that the as-deposited CoZr structured film possesses in-plane uniaxial anisotropy weakly. This was induced by uniaxial stress induced due to gradient sputtering [27]. Hysteresis loops of the easy magnetization direction were substantially a rectangle, while remanence ratio (*M*_r_*/M*_s_) was close to 1. Moreover, the difference between easy and hard axis loops increased with the increase of oblique sputtering angle, which indicated change of magnetic anisotropy.

**Figure 3:**
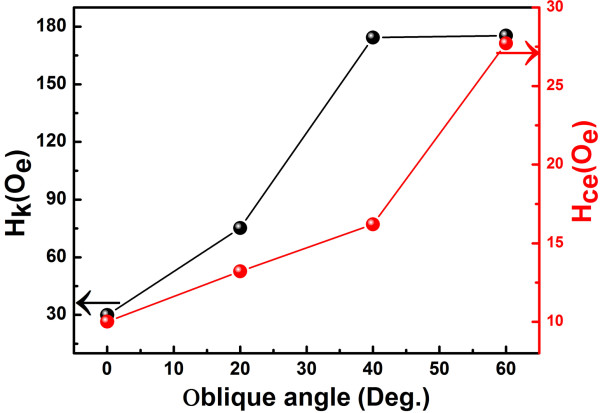
The static anisotropy effective field and the coercivity versus the oblique sputtering angle.

The overall dependences of anisotropy field *H*_k_ and coercivity of easy axis direction with various oblique sputtering angles were summarized in Figure 3. Here, *H*_k_ could be estimated by checking the cross point of the central line of the hard axis loop with the counter extension of the magnetization saturation line [28]. With increasing oblique sputtering angle, the coercivity in the easy axis (*H*_ce_) increased slightly from 10 to 27 Oe. In addition, the coercivity of nanostructure films was larger than that of continuous films [18,29], which was attributed to the change in the interaction of shape anisotropy and inhomogeneous magnetization rotation caused by the nanohill pattern of the magnetic films. As the angle increased, *H*_k_ increased monotonically, which was attributed to anisotropy induced by gradient sputtering and oblique sputtering. With increasing oblique sputtering angle, anisotropy induced by oblique sputtering was increased and played a dominant role gradually. Therefore, *H*_k_ increased with increasing oblique sputtering angle.

**Figure 4:**
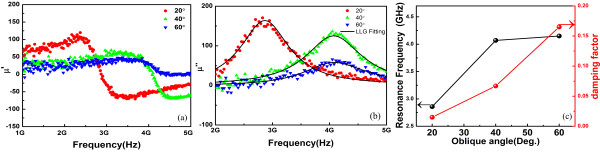
**Dependence of complex permeability *μ = μ’ − j μ” *on frequency for the films with different oblique sputtering angles.** Permeability spectra: the experimental results (symbols) and the fitting results by LLG equation (solid lines). **(a)***μ*’; **(b)***μ”*. **(c)** Resonance frequency and damping factor versus oblique sputtering angle.

Figure 4 shows the dependence of complex permeability *μ = μ’ − j μ”* on frequency for the films with different oblique sputtering angles measured by microstrip method using a vector network analyzer (PNA E8363B). The *μ’* and *μ”* represent the real and imaginary part of complex permeability. Due to weak magnetic anisotropy in the sample with an oblique sputtering angle of 0°, the curve of complex permeability depending on frequency was almost unchanged. Hence, the data was not included here. From Figure 4b, the peak of the imaginary complex permeability shifted to high frequency with increasing oblique sputtering angle. Furthermore, the linewidth of all samples was above 1 GHz, which was larger compared with that of continuous films at around 0.5 GHz [30]. Generally, the permeability spectra could be analyzed based on the LLG equation [5]; based on Equation 1, the permeability spectrum of magnetic thin film with in-plane uniaxial anisotropy can be expressed as [31]

(3) $$\mu =1+\frac{\omega_{m}\left(\omega_{0}+\omega_{m}+\mathrm{i\alpha \omega}\right)}{\omega_{r}^{2}-\omega^{2}+\mathrm{i\alpha \Delta}\omega_{r}}\;$$

with *ω*_*m*_ *= γ*4*πM*_s_*, ω*_0_ *= γH*_k_*, ω*^2^_*r*_ *= ω*^2^_0_ *+ ω*_0_*ω*_*m*_*, Δω*_*r*_ *= α*(2*ω*_0_ *+ ω*_*m*_*)*, where 4π*M*_s_ is the saturation magnetization and takes the measurement value.

The permeability spectrum can be fitted with Equation 3, as shown by the solid lines in Figure 4b. The fitting parameters are plotted in Figure 4c. The resonance frequency (*f*_r_) increased from 2.9 to 4.2 GHz with the increase of oblique sputtering angle, which had the same tendency with that of *H*_k_. The damping factor also increased from 0.015 to 0.165, which was larger than that of continuous films at around 0.01 [30]. Intrinsic damping and extrinsic sample inhomogeneities were two dominant contributions to the linewidth. The intrinsic LLG damping was generally a confluent process such as magnon-electron scattering. There was also extrinsic damping via two-magnon processes, such as the result of scattering from grain and grain boundaries, etc. Both the intrinsic and extrinsic processes lead to loss in the system. Besides the above two factors, an additional source of the linewidth was the sample inhomogeneities (not a real loss) which typically resulted in the distribution of material properties, such as the anisotropy, that would increase the linewidth. In order to understand the origin of the enhancement of the linewidth and/or damping factor, FMR was measured as a function of the angle between external magnetic field and in-plane easy axis.

The ferromagnetic resonance equation for out-of-plane measurement configuration [32] is given as follows:

(4) $$\begin{array}{l} {\left(\frac{\omega }{\gamma }\right)}^{2}=\left\{H\cos\left(\theta_{M}-\theta_{H}\right)-\left(4\pi M_{s}-\frac{2K_{⊥}}{M_{s}}\right)\cos2\theta_{M}\right\}\\ \;\times \left\{H\cos\left(\theta_{M}-\theta_{H}\right)-\left(4\pi M_{s}-\frac{2K_{⊥}}{M_{s}}\right)\cos^{2}\theta_{M}-\frac{2K_{u}}{M_{s}}\right\}\; \end{array}$$

where *γ* is the gyromagnetic ratio, 4π*M*_s_ is the saturation magnetization of the film, *K*⊥ is the perpendicular magnetic anisotropy constant, *θH* is the angle between the external field and film normal, and *θM* is the angle between magnetization vector and film normal.

**Figure 5:**
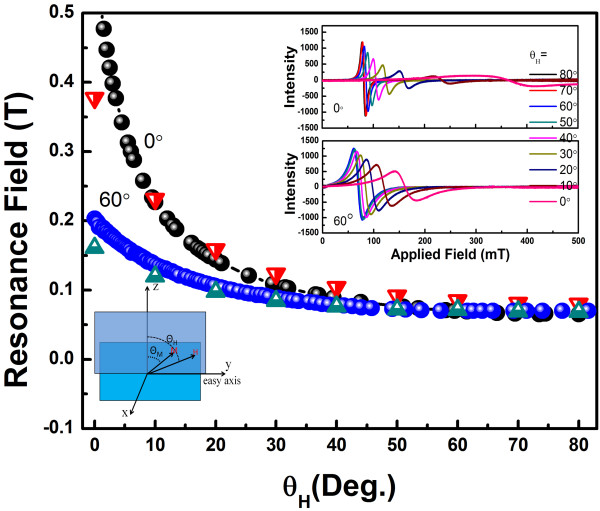
**Resonance field versus the angle between the external field and the easy axis.** The circles represent the resonance fields for CoZr films deposited at a tilted substrate angle of 0° and 60°, respectively. The triangles are theoretical lines obtained by Equation 5. The insets are ESR of the samples with oblique sputtering angle of 0° and 60°.

The measurement configuration was shown in the inset of Figure 5. The out-of-plane resonance field versus field orientation *θH* for films deposited at an oblique sputtering angle of 0° and 60° is shown in Figure 5. The resonance fields decreased monotonically for each film with increasing angle between the external field *H* and the film normal, which was caused by the demagnetization energy when the external field *H* was parallel to film normal. Moreover, the magnitude of resonance field decreased with increasing oblique sputtering angle, which was closely related to the perpendicular anisotropy field 2*K*⊥/*M*_s_ in the first term on the right side of Equation 4. Taking into account the equilibrium equation of magnetization

(5) $$H\sin\left(\theta M0-\mathrm{\theta H}\right)-\left(4\pi M_{s}-2K⊥/M_{s}\right)\sin\theta_{M0}\cos\theta_{M0}=0$$

Here the saturation magnetization 4*πM*_s_ was obtained by static VSM measurement; the perpendicular magnetic anisotropy constant could be acquired by fitting the experimental data with Equation 5. The fitted result showed that *K*⊥ of 60° was 16.3 × 10^3^ erg/cm_3_ larger than the 12.9 × 10^3^ erg/cm^3^ of 0°, which indicated increase with increasing oblique sputtering angle. Generally, the *K*⊥ of continuous film was almost zero due to strong demagnetization energy. In our case, the decrease of demagnetization energy was caused by shape anisotropy of nanostructure films, which induced the increase of *K*⊥. Therefore, the increase of *K*⊥ induced inhomogeneities of magnetic anisotropy, which resulted in the increase of linewidth and/or damping factor.

## Conclusions

The static and dynamic magnetic properties of CoZr/AAO films with different oblique sputtering angles have been investigated. All the properties and parameters were found to be dependent on magnetic anisotropy field which was induced by the shape of the AAO template and oblique sputtering. The competition between the two factors resulted in the trend of dependence on anisotropy field *H*_k_ and remanence ratio *M*_r_*/M*_s_, with various oblique sputtering angles. The resonance frequency change of CoZr/AAO films was also attributed to the effect of properties and oblique sputtering. Enhanced microwave absorption was confirmed by complex permeability measurement comparing with continuous film on a Si substrate.

## Abbreviations

AAO: Anodized aluminum oxide; AFM: Atomic force microscope; SEM: Scanning electron microscope; VSM: Vibrating sample magnetometer; FMR: Ferromagnetic resonance.

## Competing interests

The authors declare that they have no competing interests.

## Authors’ contributions

FW fabricated the CoZr films, performed the measurements, and wrote the manuscript. CJ analyzed the results and wrote the manuscript. GW helped to grow and measure the films. DX supervised the overall study. All authors read and approved the final manuscript.
